# Multidimensional Proteome Profiling of Blood-Brain Barrier Perturbation by Group B *Streptococcus*

**DOI:** 10.1128/mSystems.00368-20

**Published:** 2020-08-25

**Authors:** Anaamika Campeau, Robert H. Mills, Marie Blanchette, Kaja Bajc, Mario Malfavon, Roeben N. Munji, Liwen Deng, Bryan Hancock, Kathryn A. Patras, Joshua Olson, Victor Nizet, Richard Daneman, Kelly Doran, David J. Gonzalez

**Affiliations:** a Department of Pharmacology, University of California, San Diego, La Jolla, California, USA; b Skaggs School of Pharmacy and Pharmaceutical Sciences, University of California, San Diego, La Jolla, California, USA; c Center for Microbiome Innovation, University of California, San Diego, La Jolla, California, USA; d Department of Neuroscience, University of California, San Diego, La Jolla, California, USA; e Division of Host-Microbe Systems and Therapeutics, Department of Pediatrics, University of California, San Diego, La Jolla, California, USA; f Collaborative to Halt Antibiotic-Resistant Microbes, University of California, San Diego, La Jolla, California, USA; g Department of Immunology and Microbiology, University of Colorado, Aurora, Colorado, USA; Northern Arizona University

**Keywords:** group B *Streptococcus*, TMT, blood-brain barrier, meningitis, multiplexing, proteomics

## Abstract

Group B *Streptococcus* (GBS) remains the leading cause of neonatal meningitis, a disease associated with high rates of adverse neurological sequelae. The *in vivo* relationship between GBS and brain tissues remains poorly characterized, partly because past studies had focused on microbial rather than host processes. Additionally, the field has not capitalized on systems-level technologies to probe the host-pathogen relationship. Here, we use multiplexed quantitative proteomics to investigate the effect of GBS infection in the murine brain at various levels of tissue complexity, beginning with the whole organ and moving to brain vascular substructures. Infected whole brains showed classical signatures associated with the acute-phase response. In isolated brain microvessels, classical blood-brain barrier proteins were unaltered, but interferon signaling and leukocyte recruitment proteins were upregulated. The choroid plexus showed increases in peripheral immune cell proteins. Proteins that increased in abundance in the vasculature during GBS invasion were associated with major histocompatibility complex (MHC) class I antigen processing and endoplasmic reticulum dysfunction, a finding which correlated with altered host protein glycosylation profiles. Globally, there was low concordance between the infection proteome of whole brains and isolated vascular tissues. This report underscores the utility of unbiased, systems-scale analyses of functional tissue substructures for understanding disease.

**IMPORTANCE** Group B *Streptococcus* (GBS) meningitis remains a major cause of poor health outcomes very early in life. Both the host-pathogen relationship leading to disease and the massive host response to infection contributing to these poor outcomes are orchestrated at the tissue and cell type levels. GBS meningitis is thought to result when bacteria present in the blood circumvent the selectively permeable vascular barriers that feed the brain. Additionally, tissue damage subsequent to bacterial invasion is mediated by inflammation and by immune cells from the periphery crossing the blood-brain barrier. Indeed, the vasculature plays a central role in disease processes occurring during GBS infection of the brain. Here, we employed quantitative proteomic analysis of brain vascular substructures during invasive GBS disease. We used the generated data to map molecular alterations associated with tissue perturbation, finding widespread intracellular dysfunction and punctuating the importance of investigations relegated to tissue type over the whole organ.

## Abstract (precis)

Group B *Streptococcus* (GBS) meningitis remains a major cause of poor health outcomes very early in life. Both the host-pathogen relationship leading to disease and the massive host response to infection contributing to these poor outcomes are orchestrated at the tissue and cell type levels. GBS meningitis is thought to result when bacteria present in the blood circumvent the selectively permeable vascular barriers that feed the brain. Additionally, tissue damage subsequent to bacterial invasion is mediated by inflammation and by immune cells from the periphery crossing the blood-brain barrier. Indeed, the vasculature plays a central role in disease processes occurring during GBS infection of the brain. Here, we employed quantitative proteomic analysis of brain vascular substructures during invasive GBS disease. We used the generated data to map molecular alterations associated with tissue perturbation, finding widespread intracellular dysfunction and punctuating the importance of investigations relegated to tissue type over the whole organ.

## INTRODUCTION

Streptococcus agalactiae, or group B *Streptococcus* (GBS) remains the leading cause of neonatal sepsis and meningitis (1). Notably, up to 50% of infants that recover from GBS meningitis suffer from neurological problems, such as blindness, deafness, and cerebral palsy, later in life (2, 3). It has been posited that perturbation of the system of continuous capillaries that supply the brain tissues with nutrients, collectively known as the blood-brain barrier (BBB), is a prerequisite for the development of GBS meningitis (4, 5). Despite decades of study, the relationship between GBS and tissues of the BBB remains poorly understood.

The capillaries of the BBB are characterized by the maintenance of specialized cell-cell junctions and by low rates of transcytosis that prevent the unimpeded passage of cells and molecules from the blood into the privileged tissues of the brain (6). Also important to the protection of the brain tissues from the blood is the choroid plexus (CP), a fenestrated vascular structure in the brain that comprises a part of the blood-cerebrospinal fluid (CSF) barrier. Several studies have investigated GBS interactions with the BBB by studying specific bacterial virulence factors (5, 7–13). Among these was a study identifying *iagA*, encoding a putative lipoteichoic acid (LTA) anchor that was critical for penetration of brain microvascular endothelial cells (BMECs) *in vitro* and for GBS meningitis *in vivo* (14). However, fewer studies have investigated changes to the host during this disease. Additionally, to our knowledge, no studies have taken advantage of systems-level techniques such as proteomics to unbiasedly evaluate changes in these important physiological barriers during active GBS infection.

While bacterial perturbation of the BBB precedes GBS meningitis, damage to the central nervous system (CNS) tissues associated with meningitis is largely the result of the host response to the presence of bacteria within the brain. Indeed, a study on MyD88^−/−^ and TLR2^−/−^ mice demonstrated that the mutants lacking these key immune proteins had better overall outcomes than wild-type (WT) mice upon GBS infection (15). Host-centered studies can also shed light on pathogenic processes. One study showed that the presence of GBS was sufficient to activate the autophagy pathway in brain microvascular endothelial cells both *in vitro* and *in vivo* (16), suggesting that GBS perturbs intracellular functions in the BBB endothelium. Additional studies on responses to GBS infection of the brain tissues can improve our understanding of host-pathogen interactions and the contribution of the immune response to clinical outcomes.

Our goals in this study were 2-fold. First, we sought to understand the tissue-specific proteome signatures underpinning the host-pathogen relationship in GBS meningitis. Second, we endeavored to unbiasedly characterize molecular changes to the BBB during this disease, including posttranslational modifications (PTM) of the host proteome. We achieved these goals by performing quantitative mass spectrometry (MS)-based proteomic analysis of whole brains, brain capillaries, and choroid plexi collected from animals infected with a WT virulent GBS strain and its isogenic invasion-deficient mutant strain (Δ*iagA*). While whole brains showed changes in acute-phase reactants typical of systemic bacterial infection, specific evaluations of the brain vasculature yielded a more granular view into the alterations associated with bacterial perturbation of the BBB. Proteomic data from isolated microvessels and choroid plexi allowed us to map protein pathway changes in the CNS vasculature during infection, identifying several innate immune-related changes in both tissues. We also found invasion-associated increases in endogenous antigen presentation machinery and endoplasmic reticulum (ER) stress response proteins. Conversely, there were no significant changes observed in many classical molecular features of the BBB. Given the established relationship between ER dysfunction and protein glycosylation, we expanded our search parameters to identify changes in posttranslational modifications occurring during infection, determining that invasive GBS infection was strongly associated with altered glycosylation of several secreted and cell surface proteins. Our study yielded proteome signatures of GBS infection in the brain vasculature not detectable through a whole-organ approach, providing an important snapshot into molecular pathway changes during GBS meningitis.

## RESULTS

### BBB proteome mapping of the brain tissues during GBS infection.

**FIG 1:**
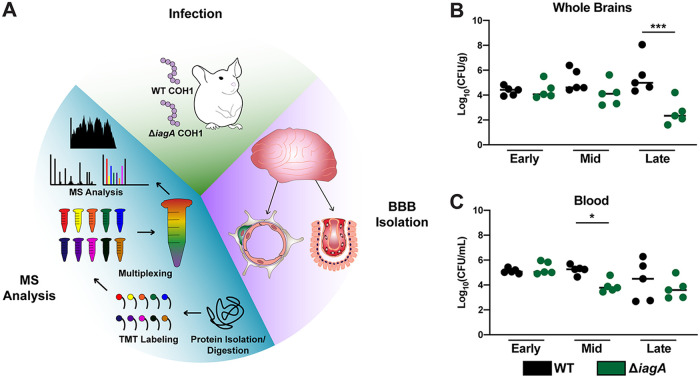
Experimental outline showing strategy for delineating infection- and invasion-associated proteome changes during GBS meningitis. (A) Schematic detailing experimental strategy for mouse infection with WT and Δ*iagA* GBS isogenic strains, vascular tissue isolation, and proteomic analysis of isolated substructures. (B and C) CFU enumeration of whole-brain tissue (B) and blood tissue (C) collected from mice infected with WT and Δ*iagA* COH1 GBS at 16, 38, and 62 h postinfection. Statistical significance was determined through one-way ANOVA (*, *P* < 0.05; ***, *P* < 0.001).

To assess the proteome changes in the brain tissues occurring during GBS infection, mice were first infected with WT GBS strain COH1, a virulent encapsulated serotype III strain, and its isogenic Δ*iagA* mutant (14). To assess the differences in infectivity of brain tissues in WT and Δ*iagA* strains at various time points, animals were sacrificed at 16 h, 38 h, and 62 h postinfection. It was noted that at the 62-h time point, WT GBS-infected mice demonstrated signs of meningitis, such as seizures and motor difficulties. Whole-brain and blood tissues were harvested and subjected to homogenization for CFU enumeration (*n* = 5) (Fig. 1B and C). Counts of CFU recovered from brain tissue were significantly higher in the WT strain than in the Δ*iagA* strain at the latest time point (62 h postinfection). Counts of CFU recovered from the blood were not significantly different between the two strains at this time point, supporting past research indicating that the two strains differ in their ability to mediate disease in the brain tissues (14).

The whole-brain tissue was next subjected to tandem mass tag (TMT)-based quantitative multiplexed proteomic analysis, with 8,563 proteins quantified in total and 3,245 proteins quantified in common among all multiplexed experiments (*n* = 4 to 5). Binary comparisons between WT- and mock-infected animals were performed to define the set of proteins significantly altered in abundance during GBS infection of the brain tissues (see Data Set S1 and Fig. S1A to C in the supplemental material). Of the differentially abundant proteins, a core set of 11 proteins were increased in abundance at all time points, while only one protein was decreased in abundance at all time points (Fig. S1D and E). Among the common proteins that were increased in abundance, all (Saa1, Lrg1, Hpx, Hp, Itih4, Serpina3n, Fga/b/g, Cp, and C3) were associated with the acute-phase response to infection (Fig. S1E). Ttr, a thyroxine transport protein previously identified in both serum and cerebrospinal fluid (CSF), was decreased in abundance at all time points (17). Although these changes aligned with known features of bacterial sepsis and meningitis, the use of whole-brain tissue analysis in this experiment precluded the tissue-specific localization of these signals to the vascular barriers between the blood and brain.

10.1128/mSystems.00368-20.1 FIG S1 CFU enumeration and quantitative proteomic analysis of whole brains infected with GBS. (A to C) Binary comparisons of WT GBS-infected whole brains. Colored dots represent significantly upregulated (red) or downregulated (blue) proteins in WT GBS-infected brains compared to mock-infected brains (π > 1). (D) Venn diagram showing overlap of significantly altered proteins in WT GBS-infected compared to mock-infected whole brains at each time point. (E) Average protein abundance values for significantly upregulated and downregulated proteins common to all time points in WT GBS-infected compared to mock-infected whole brains at each time point. (F) Overlap of all identified proteins in vessel and choroid plexus proteomics experiments. (G) Tree map representing reactome functional enrichments for proteins uniquely identified in vessels. Cells are sized by −Log_10_(*P* value) data representing enrichment. (H) Tree map representing reactome functional enrichments for proteins uniquely identified in choroid plexi. Cells are sized by −Log_10_(*P* value) data representing enrichment. Download FIG S1, PDF file, 0.9 MB. Copyright © 2020 Campeau et al. 2020 Campeau et al. This content is distributed under the terms of the Creative Commons Attribution 4.0 International license.

10.1128/mSystems.00368-20.6 DATA SET S1 Whole-brain proteome data from WT GBS-, Δ*iagA* GBS-, and mock-infected CD-1 mice sacrificed 16 (early), 38 (mid), and 62 (late) hours postinfection. Sample column headers are labeled using the following scheme: infection status_infection strain_timepoint. The labels and contents of the data set sheets are as follows: ALL, all proteins detected and quantified across all whole-brain multiplexed experiments; COMMON, proteins detected and quantified in common across all whole-brain multiplexed experiments; WTvU_16, binary calculations from comparisons between whole-brain proteome data collected from WT GBS-infected animals and from mock-infected animals sacrificed at 16 h postinfection (proteins are segregated by pi scores of >1 and ranked by fold change); WTvU_38, binary calculations from comparisons between whole-brain proteome data collected from WT GBS-infected animals and from mock-infected animals sacrificed at 38 h postinfection (proteins are segregated by pi scores of >1 and ranked by fold change); WTvU_62: binary calculations from comparisons between whole-brain proteome data collected from WT GBS-infected animals and from mock-infected animals sacrificed at 62 h postinfection (proteins are segregated by pi scores of >1 and ranked by fold change); Wtviag_PBSnorm_16, binary calculations from comparisons between whole-brain proteome data collected from WT GBS-infected and from Δ*iagA* GBS-infected animals sacrificed at 16 h postinfection normalized against average mock-infected protein abundance values (proteins are segregated by pi scores of >1 and ranked by fold change); Wtviag_PBSnorm_38, binary calculations from comparisons between whole-brain proteome data collected from WT GBS-infected and from Δ*iagA* GBS-infected animals sacrificed at 38 h postinfection normalized against average mock-infected protein abundance values (proteins are segregated by pi scores of >1 and ranked by fold change); Wtviag_PBSnorm_62, binary calculations from comparisons between whole-brain proteome data collected from WT GBS-infected and from Δ*iagA* GBS-infected animals sacrificed 62 h postinfection normalized against average mock-infected protein abundance values (proteins are segregated by pi scores of >1 and ranked by fold change). Download Data Set S1, XLSX file, 13.1 MB. Copyright © 2020 Campeau et al. 2020 Campeau et al. This content is distributed under the terms of the Creative Commons Attribution 4.0 International license.

To address the shortcomings of whole-organ proteomics in a disease-relevant tissue type, a separate cohort of mice was infected with WT and Δ*iagA* GBS, and all animals were sacrificed 62 h postinfection. Capillaries (*n* = 6 per infection group) and choroid plexi (*n* = 3 to 4 per infection group) were isolated from the brains of infected mice and subjected to multiplexed quantitative proteomic analysis (Fig. 1A). For vessel proteomics, we detected and quantified 4,786 proteins in total and 3,305 proteins in common between the two multiplexed experiments and we detected 4,867 proteins in the choroid plexus experiment (Data Set S2 and S3, respectively). On the basis of these numbers, this study was among the most comprehensive proteomic analyses of *in vivo* blood-brain barrier and choroid plexus tissues performed to date (18–30). Among the proteins detected in each of these tissue types, 3,051 were common to both, while 1,734 and 1,816 were unique to the vessels and choroid plexus, respectively (Fig. S1F). Reactome pathway analysis was performed on the proteins unique to each tissue type, where the most significantly enriched terms were “Axon Guidance” for vessels and “TNF Signaling” for the choroid plexus (Fig. S1G and H) (31). These findings underscore the role of differential protein abundance in dictating the functional characteristics of each of these two tissue types.

10.1128/mSystems.00368-20.7 DATA SET S2 Brain microvasculature proteome data from WT GBS-, Δ*iagA* GBS-, and mock-infected CD-1 mice sacrificed 62 h postinfection. The labels and contents of the data set sheets are as follows: ALL, all proteins detected and quantified across all brain microvasculature multiplexed experiments; COMMON, proteins detected and quantified in common across all brain microvasculature multiplexed experiments; WTvU, binary calculations from comparisons between brain microvasculature proteome data collected from WT GBS-infected animals and from mock-infected animals (proteins are segregated by pi scores of >1 and ranked by fold change); iagvU, binary calculations from comparisons between whole-brain proteome data collected from Δ*iagA* GBS-infected animals and from mock-infected animals (proteins are segregated by pi scores of >1 and ranked by fold change; WTviag, binary calculations from comparisons between brain microvasculature proteome data collected from WT GBS-infected and from Δ*iagA* GBS-infected animals normalized against average mock-infected protein abundance values (proteins are segregated by pi scores of >1 and ranked by fold change). Download Data Set S2, XLSX file, 6.6 MB. Copyright © 2020 Campeau et al. 2020 Campeau et al. This content is distributed under the terms of the Creative Commons Attribution 4.0 International license.

10.1128/mSystems.00368-20.8 DATA SET S3 Choroid plexus proteome data from WT GBS-, Δ*iagA* GBS-, and mock-infected CD-1 mice sacrificed 62 h postinfection. The labels and contents of the data set sheets are as follows: ALL, all proteins detected and quantified across choroid plexus multiplexed experiment; WTvU, binary comparison between WT GBS-infected and uninfected choroid plexus data (proteins are segregated by pi scores of >1 and ranked by fold change); iagvU, binary comparison between Δ*iagA* GBS-infected and uninfected choroid plexus data (proteins are segregated by pi scores of >1 and ranked by fold change); WTviag, binary calculations from comparisons between choroid plexus proteome data collected from WT GBS-infected and from Δ*iagA* GBS-infected animals normalized against average mock-infected protein abundance values (proteins are segregated by pi scores of >1 and ranked by fold change). Download Data Set S3, XLSX file, 5.8 MB. Copyright © 2020 Campeau et al. 2020 Campeau et al. This content is distributed under the terms of the Creative Commons Attribution 4.0 International license.

### Classical molecular signatures of the blood-brain barrier are unchanged during GBS meningitis.

**FIG 2:**
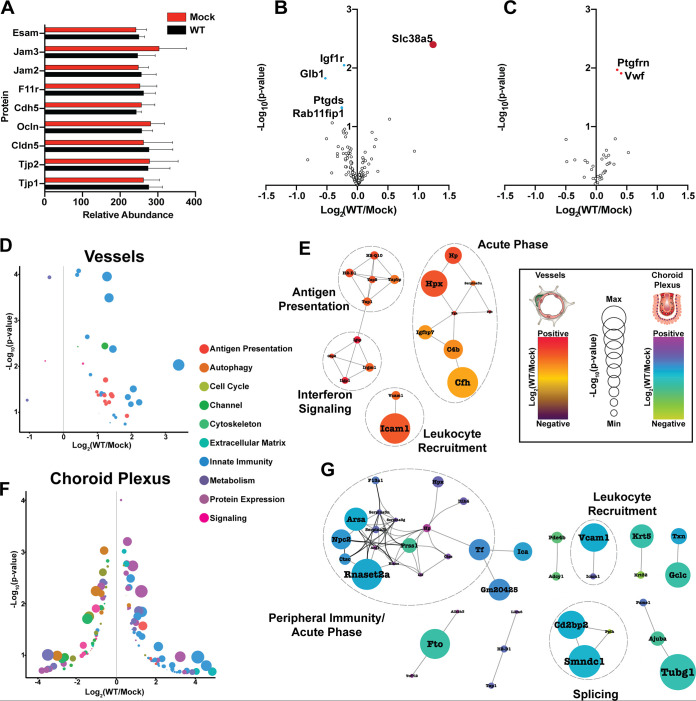
GBS infection is associated with changes in immune-related proteins in the brain vasculature. (A) Relative-abundance values of classical blood-brain barrier protein markers in comparison between WT GBS- versus mock-infected blood vessels. (B) Comparison of previously published blood-brain barrier proteins from WT GBS-infected versus uninfected brain vessels (dark red, π score > 1; light blue, *P* value < 0.05; Student's *t* test). (C) Comparison of previously published blood-brain barrier dysfunction proteins from WT GBS-infected versus uninfected brain vessels (light red, *P* < 0.05; Student's *t* test). (D) Bubble plot demonstrating the functional annotations of significantly altered proteins in a comparison of WT GBS versus uninfected brain vessels (π score > 1). (E) Protein-protein interaction networks for significantly altered proteins in WT GBS-infected versus uninfected brain vessels (π score > 1). Networks were generated using String-db associations (interaction score > 0.9). (F) Bubble plot demonstrating the functional annotations of significantly altered proteins in a comparison of WT GBS versus uninfected choroid plexi (π score > 1). (G) Protein-protein interaction networks for significantly altered proteins in WT GBS-infected versus uninfected choroid plexi (π score > 1). Networks were generated using String-db associations (interaction score > 0.9).

In crossing from the blood into the brain tissues, GBS is thought to circumvent the BBB, thus provoking inflammation and damage to the CNS. Therefore, we evaluated the brain vasculature proteome data for abundance changes in classical proteins implicated in BBB integrity during GBS infection. Comparing WT COH1-infected versus mock-infected vessels, we saw no significant difference in the abundances of the cell-cell junction proteins known to confer characteristic continuity to the brain microvasculature, including Tjp1, Tjp2, Cldn5, Ocldn, Cdh5, JAM2, JAM3, F11r, and Esam (6, 32, 33) (Fig. 2A). We next contextualized our data against a published data set of BBB-specific genes (34). Of 517 genes deemed “BBB-enriched” through transcriptomic profiling, we detected 122 in our proteomics data set. Altered proteins were identified using π scores, a metric that accounts for both statistical significance and fold change. We evaluated protein abundance changes during infection with WT GBS for these 122 proteins and found that only a single protein exceeded a π score of 1 (increased abundance; Slc38a5), with 5 proteins exceeding a *P* value-based significance threshold (decreased abundance, Igf1r, Glb1, Ptgds, and Rab11fip1; increased abundance, Slc38a5) (*P* < 0.05) (Fig. 2B). We next attempted to benchmark infection-dependent changes in the BBB against gene expression changes reported in a previously published BBB dysfunction gene module (34). Of the 136 genes found to be strongly associated with BBB dysfunction in that study, we detected 35 in our proteomics data set. Evaluating GBS infection-dependent changes, we found that no proteins exceeded a π score of 1 and that only 2 proteins (increased abundance; Ptgfrn and Vwf) showed significantly different abundances using a *P* value-based significance threshold (*P* < 0.05) (Fig. 2C). Taken together, these findings suggest that many classical features of the BBB are maintained during GBS meningitis and that perturbation of the BBB during GBS meningitis is distinct from other forms of brain injury where BBB dysfunction has been implicated.

### GBS infection is associated with changes in immune-related proteins in the brain vasculature.

We next assessed the infection-dependent changes occurring in the BBB during GBS infection, performing binary comparisons on the vessel and choroid plexus data collected from WT COH1-infected and mock-infected animals. Vessel proteins meeting a threshold of a π score of >1 were subjected to functional annotation (Fig. 2D; see also Data Set S2), and the majority of changes identified in the brain vasculature were related to the host immune response. Among the most highly significant changes in the brain microvasculature were those seen with Ackr1, Icam1, and Vcam1. These proteins play important roles in the recruitment of peripheral immune cells to the brain tissue during various neurological diseases (35–41). This analysis also detected an increase in the abundance of transport proteins Slc38a5 and Slc12a7. Both transporters have been identified as important components of the BBB (34, 42, 43), but their function during BBB perturbation remains poorly characterized.

Protein interaction networks were constructed to identify groups of proteins influenced by GBS infection. Analysis of the vessel data revealed several proteins related to the acute-phase response to infection (Hp, Hpx, Serpina3n, Fga/b, Igfbp7, Cfh, and C4b), antigen presentation (Tap1, Tap2, Tapbp, H2-D1, and H2-Q10), and interferon response (Igtp, Gbp2, Irgm1, and Iigp1) (Fig. 2E). Binary comparisons of WT GBS-infected and uninfected choroid plexus tissue revealed many differentially regulated proteins (Fig. 2F; see also Data Set S3). Network analysis of these proteins revealed an increase in the abundance of proteins related to innate immune processes and the acute-phase response (Arsa, Npc2, Ctsc, Rnaset2a, Elane, Arg1, Serpina3i/g/n, F13a1, Prss1, Ltf, Ctss, Hp, Hpx, Itih4, Tf, Ica, and Gm20425) (Fig. 2G). This is consistent with hallmark clinical features of GBS meningitis, in particular, massive leukocyte infiltration into the CSF (44).

Finally, we evaluated the degree to which infection-dependent changes in the brain microvessels and choroid plexus could be identified in the whole-brain proteomics data. We identified 1,896 proteins in common between the whole brains and microvessels and 1,645 shared between the whole brains and choroid plexi. There was low concordance between WT infection-dependent changes in whole brains and those in microvessels (*r* = 0.0833) and choroid plexi (*r* = 0.1197) (Fig. S2B and E, respectively). Low concordance was also found between invasion-dependent changes in whole brains compared to microvessels (*r* = −0.0663) and choroid plexi (*r* = 0.0941) (Fig. S2D and F). These findings highlight the potential value of our tissue type-based strategy for probing the proteomics of GBS interaction with BBB structures.

10.1128/mSystems.00368-20.2 FIG S2 Proteome abundance overlay of microvessel and choroid plexus compared to whole brains. (A) Overlap of identified proteins in brain microvessel proteomics experiment and whole-brain proteomics experiment. (B) Correlation plot of Log_2_(fold change) between WT GBS-infected and uninfected brain microvessels and whole brains at 62 h postinfection. (C) Correlation plot of Log_2_(fold change) between WT GBS-infected and Δ*iagA* GBS-infected brain microvessels and whole brains at 62 h postinfection. (D) Overlap of identified proteins in choroid plexus proteomics experiment and whole-brain proteomics experiment. (E) Correlation plot of Log_2_(fold change) between WT GBS-infected and uninfected choroid plexi and whole brains at 62 h postinfection. (F) Correlation plot of Log_2_(fold change) between WT GBS-infected and Δ*iagA* GBS-infected choroid plexi and whole brains at 62 h postinfection. Download FIG S2, PDF file, 0.8 MB. Copyright © 2020 Campeau et al. 2020 Campeau et al. This content is distributed under the terms of the Creative Commons Attribution 4.0 International license.

### Invasive GBS infection in the brain vasculature results in increased intracellular stress proteins.

**FIG 3:**
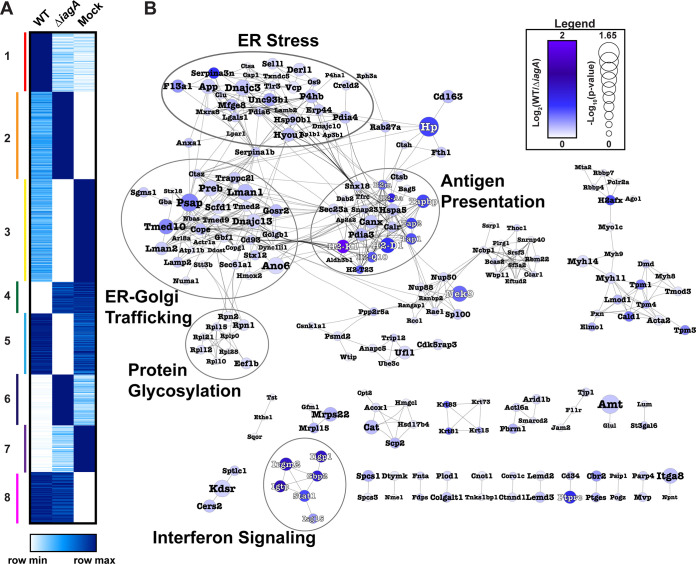
Mapping invasion-dependent proteome changes reveals altered endoplasmic reticulum protein pathway abundances. (A) Heat map of k-means clustered vessel proteomics average values from WT GBS-infected, Δ*iagA* GBS-infected, and mock-infected animals. To the left of the heat map, clusters are numbered and delineated with colored bars. (B) Protein interaction network of invasion-associated cluster 1 (interaction score > 0.9). Nodes are sized by significance of the comparison between WT GBS-infected versus Δ*iagA* GBS-infected vessels. Nodes are colored by fold change between WT GBS-infected and Δ*iagA* GBS-infected quantitation value averages. Functional clusters are circled and labeled. Node labels are colored to enhance visibility against the background. Nodes are sized by −Log_10_(*P* value), with increased significance associated with increased node size.

Because previous studies showed that the *iagA* gene was associated with GBS invasion into the brain tissues, we next explored proteins with different abundance profiles in the brain vasculature during WT GBS-infected and Δi*agA* GBS infection. To identify disease-relevant protein abundance changes, we performed k-means clustering on the average values corresponding to each protein in WT GBS, Δi*agA* GBS, and mock infection (Fig. 3A; see also Data Set S4). Each cluster was then subjected to functional enrichment analysis using the KEGG search function of g:Profiler (Fig. S3). This analysis revealed a group of proteins (cluster 1) whose abundance was increased during infection with the WT strain but unchanged during infection with the Δi*agA* strain (Fig. S3A). The most strongly enriched terms associated with cluster 1 were “Protein Processing in ER,” “Antigen Processing and Presentation,” and “Phagosome.”

10.1128/mSystems.00368-20.3 FIG S3 Functional GO analysis of all clusters derived from k-means clustering of brain microvessel proteomics data. (A to H) Bar graphs representing significantly enriched functional terms from GO analysis of brain microvessel protein clusters derived from k-means clustering (8 clusters). Violin plot insets demonstrate the within-cluster average protein abundance trends for each experimental group. Download FIG S3, PDF file, 2.2 MB. Copyright © 2020 Campeau et al. 2020 Campeau et al. This content is distributed under the terms of the Creative Commons Attribution 4.0 International license.

10.1128/mSystems.00368-20.9 DATA SET S4 K-means protein clusters. Download Data Set S4, XLSX file, 0.1 MB. Copyright © 2020 Campeau et al. 2020 Campeau et al. This content is distributed under the terms of the Creative Commons Attribution 4.0 International license.

The proteins belonging to this cluster were further analyzed for functional groupings through network analysis (confidence score > 0.9) (Fig. 3B). The networks identified clusters related to endoplasmic reticulum (ER) stress, ER-Golgi trafficking, and protein glycosylation. Of the 434 proteins identified in this cluster, 130 were associated with “very high” interaction confidence scores (30%) and with ER biology. Among the most highly differentially abundant proteins between WT and Δi*agA* GBS infection were proteins related to endogenous antigen presentation via major histocompatibility complex (MHC) class I. In addition to MHC class I subunits, this cluster included several signaling molecules related to peptide antigen loading in the ER, such as Tap1, Tap2, Tapbp, Calr, and Canx (45).

**FIG 4:**
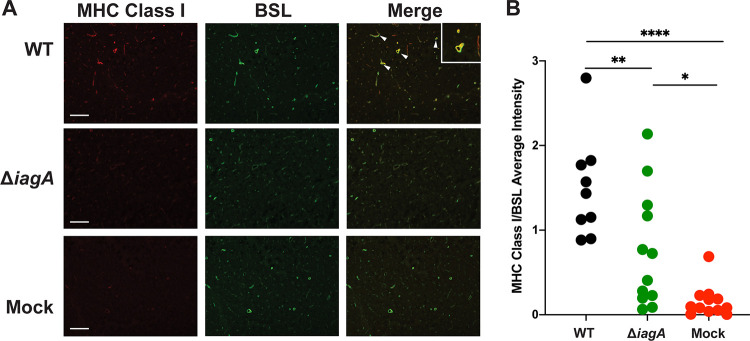
Invasive GBS infection in the brain engages vascular MHC class I antigen presentation machinery. (A) Immunofluorescent staining of whole-brain sections taken from mice infected with WT GBS or with Δ*iagA* GBS or mock infected. Sections were stained for MHC class I and BSL (a marker of blood vessels). (B) Quantitation of mean fluorescence for MHC class I staining images collected from three disparate regions of the cortex (WT infected, *n* = 3; *iagA* infected and mock infected, *n* = 4). Statistical significance was determined using one-way ANOVA (*, *P* < 0.05; **, *P* < 0.01; ****, *P* < 0.0001).

We next endeavored to validate the invasion-associated increases in MHC class I protein abundance using an alternative method. To achieve this, we performed immunofluorescent staining for MHC class I in brain tissues collected from mice injected with WT GBS, Δi*agA* GBS, or phosphate-buffered saline (PBS) (red). Bandeiraea simplicifolia lectin (BSL) stain was used to identify the brain vasculature (green). Mice infected with WT GBS showed staining for MHC class I in the brain microvasculature. In contrast, the vessels of mice either infected with Δi*agA* GBS or mock infected showed significantly weaker MHC class I staining (Fig. 4). MHC class I staining was highly restricted to the brain vasculature and was not found in other tissue types within the brain (Fig. 4A). Levels of a known marker of vascular inflammation, Vcam1, were increased above baseline during both WT and Δi*agA* GBS infection as shown by the microvessel proteomics data (Data Set S2). We validated this finding through immunofluorescent staining of whole-brain sections for Vcam1. Vcam1 staining was restricted to the brain vasculature, and the staining results showed that abundances increased during infection with both strains used in this study (Fig. S4A and B). These findings demonstrate the validity of the proteomics approach for understanding GBS invasion-dependent host proteome changes.

10.1128/mSystems.00368-20.4 FIG S4 Vcam1 abundance is increased in both WT GBS-infected and Δ*iagA* GBS-infected brain microvasculature. (A) Immunofluorescent staining validation of increased Vcam1 abundances in vasculature of WT GBS-infected and Δ*iagA* GBS-infected animals. Images were taken from the hypothalamus region of the brain in all animals. (B) Quantification of Vcam1 staining in brains of infected mice determined by normalizing average Vcam1 intensity against BSL staining. Statistical significance was determined through one-way ANOVA. Download FIG S4, PDF file, 0.9 MB. Copyright © 2020 Campeau et al. 2020 Campeau et al. This content is distributed under the terms of the Creative Commons Attribution 4.0 International license.

### Invasive GBS infection of the brain vasculature and altered glycosylation.

One of the central findings in our investigation of invasion-associated proteome changes in the brain vasculature during GBS meningitis was an increase in the abundances of proteins related to ER stress, ER-Golgi trafficking, and protein glycosylation (Fig. 3B). The ER is a major site of posttranslational glycosylation within the cell, and ER stasis has significant implications for overall cellular characteristics and cell survival. Additionally, cells alter glycosylation of proteins to enhance survival during times of ER stress (46–49). To investigate the functional impact of ER stress in the BBB during GBS infection, we assessed whether glycosylation of host proteins was altered in the brain vasculature during GBS infection.

**FIG 5:**
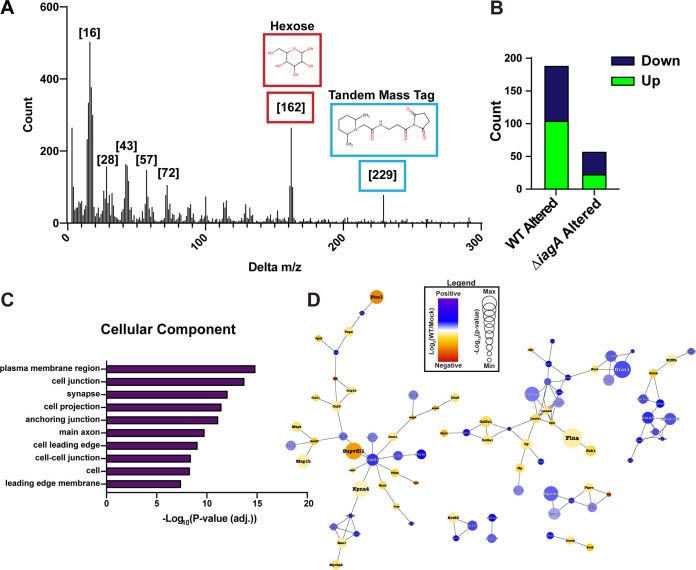
Quantitative glycoproteomics of BBB during GBS meningitis. (A) Histogram of *m*/*z* difference distribution for networked peptides derived from GNPS. Mass shifts corresponding to known chemical modifications are highlighted ([16], oxidation; [28], formylation; [43], carbamylation; [57], carbamidomethylation; [72], ethoxyformylation; [162], hexose; [229], TMT label). (B) Number of significantly altered glycopeptides in WT GBS- versus mock-infected vessels compared to the number of significantly altered glycopeptides in Δ*iagA*- versus mock-infected vessels (π score threshold > 0.5). (C) Cellular component GO analysis ranked by −Log_10_(BH-adjusted *P* value) (top 10 terms are shown). (D) String protein interaction network for significantly altered glycosylated proteins (π score threshold > 0.5; interaction threshold > 0.7). Nodes are sized by −Log_10_(*P* value), with increased significance associated with increased node size.

Utilizing Global Natural Products Social Molecular Networking (GNPS), a tool that groups spectral data in an unbiased fashion for the identification of modified peptides, we found that a 162-Da hexose mass shift was the second most abundant chemical modification in the range of 3-Da to 300-Da mass differences investigated, with a mass shift of 16 Da corresponding to oxidation found to be the most abundant (Fig. 5A). To evaluate the effect of GBS infection on glycosylation of vessel a proteins, we utilized Byonic, a proteome analysis software that enables wider search parameters to facilitate the identification of posttranslationally modified (PTM) peptides (50).

Our MS-based analysis of the mouse vessel proteome collected 515,914 tandem MS (MS/MS) spectra. However, only 120,909 spectra were matched to peptides using our standard methods, with a final match rate of 23.4%. A portion of unmatched MS/MS spectra were likely derived from heavily modified peptides, such as those that are glycosylated. In our expanded PTM-enabled search of the vessel data, we identified 180,667 peptide spectral matches (PSMs), increasing our overall match rate to 35.02% (Fig. S5A). Peptide spectral matches were compared to the corresponding MS3 spectra to yield quantitative information on 117,674 PSMs (Fig. S5B). MS3 quantitation values were summed at the glycosylated peptide level, resulting in 44,247 unique peptide features, among which 1,747 were glycosylated (Fig. S5C).

10.1128/mSystems.00368-20.5 FIG S5 Byonic analysis of spectra derived from microvessel proteomics experiment. (A) Differential peptide spectral match rates between Proteome Discoverer and Byonic. (B) Proportion of quantitative spectra matched to peptide spectral matches in Byonic analysis. (C) Count of unique peptides and unique glycosylated peptides identified from Byonic analysis. Download FIG S5, PDF file, 0.2 MB. Copyright © 2020 Campeau et al. 2020 Campeau et al. This content is distributed under the terms of the Creative Commons Attribution 4.0 International license.

Binary comparisons were performed to identify differentially abundant glycosylation events in the context of GBS infection, yielding 104 unique glycopeptides upregulated during infection (π > 0.5) (Data Set S5). In comparison to the 104 glycopeptides with increased abundance in the WT GBS-infected compared to mock-infected vessels, there were only 23 glycopeptides with increased abundance identified in our comparison of vessels collected from Δ*iagA* GBS-infected to mock-infected animals (Fig. 5B). Proteins modified in the ER are often exported to the cell surface or extracellular space. To determine the localization of the modified proteins, we performed Gene Ontology (GO) analysis of proteins with significantly altered levels of glycopeptides, focusing on cellular components. The most strongly enriched terms were “plasma membrane region,” “cell junction,” and “synapse,” indicating that many of the identified altered glycopeptides are functionally relevant to the vascular cell surface and extracellular space (Fig. S5C). We finally represented the significantly altered proteins as a functional interaction network resource in order to facilitate the further study of altered glycosylation patterns in the brain vasculature during GBS infection (Fig. 5D). In sum, these data represent the first comprehensive glycoproteomic analysis of the GBS-diseased BBB and support our hypothesis that GBS-induced ER dysfunction is associated with wide-ranging impacts on cellular function.

10.1128/mSystems.00368-20.10 DATA SET S5 Brain microvasculature Byonic peptide spectral match data from WT GBS-, Δ*iagA* GBS-, and mock-infected CD-1 mice sacrificed 62 h postinfection. The labels and contents of the data set sheets are as follows: ALL, summed unique peptide abundance values normalized against protein abundance values from Byonic; Glycosylation_WTvU, binary calculations from comparisons between WT GBS-infected and uninfected brain microvasculature unique glycopeptide quantification values (proteins are segregated by pi scores of >0.5 and ranked by fold change); Glycosylation_iagvU, binary calculations from comparisons between Δ*iagA* GBS-infected and uninfected brain microvasculature unique glycopeptide quantification values (proteins are segregated by pi scores of >0.5 and ranked by fold change). Download Data Set S5, XLSX file, 16.5 MB. Copyright © 2020 Campeau et al. 2020 Campeau et al. This content is distributed under the terms of the Creative Commons Attribution 4.0 International license.

## DISCUSSION

In the present study, we first identified protein abundance changes in BBB structures that were associated with GBS infection but were not identified in our analysis of whole brains. Among the most significantly altered proteins that we identified in the brain vasculature were those related to innate immunity and leukocyte recruitment. These findings are in line with known clinical features of GBS meningitis, including leukocyte infiltration into the brain parenchyma and associated perivascular edema. Notably, we identified a subset of BBB-related signaling proteins downregulated in the brain vasculature of infected mice, including Igf1r, Glb1, Ptgds, and Rab11fip1. Previous literature indicates that these proteins play important roles in cell signaling. Indeed, one study showed that the presence of Igf1, the ligand for Igf1r, resulted in improved BBB integrity following brain hemorrhage (51). This suggests that the altered vascular proteins identified in our study may play important roles in the pathophysiology of GBS meningitis, where the BBB is penetrated by bacteria. In the choroid plexus, we identified several proteins associated with leukocyte activity. Of note, there was only minor overlap of the altered proteins identified in whole brains and those identified in brain microvasculature and choroid plexi, underscoring the importance of tissue-specific assessments in the context of disease.

We also showed large-scale pathway changes associated with ER antigen processing, stress, and dysfunction and with invasion or persistence of bacteria in the brain. While the WT GBS-dependent increase in endogenous antigen presentation mediated via MHC class I has not previously been discussed in the literature, one study demonstrated that antibody blocking of MHC class I reduced recruitment of peripheral leukocytes during viral meningitis (52). This finding is consistent with one of the best-studied roles of MHC class I, the recruitment of immune cells to diseased tissues. Because MHC class I participates in the presentation of endogenous antigens to the peripheral immune system, this finding also suggests that GBS antigens are present in the endothelial cells that make up the BBB. Indeed, although GBS is often described primarily as an extracellular pathogen, it has been shown to enter cultured brain endothelial cells (4, 16). Additionally, numerous bacterial toxins enter host cells and exert pathogenic effects independently of bacterial entry, which might lead to both the engagement of endogenous antigen presentation and the ER dysfunction discussed below (16, 53).

We observed that several proteins related to ER stress were upregulated in an invasion-dependent manner. Previous studies have demonstrated that bacterial toxins may trigger ER stress, and some have even suggested that ER stress induced by a related species, group A *Streptococcus* (GAS), might confer a strategic advantage by allowing the bacteria greater access to amino acids as a nitrogen source (54). It is possible that GBS employs a similar strategy in its interference with cells in the BBB.

The altered glycosylation levels observed in this study could be linked to the increase in the abundance of proteins associated with ER stress and vesicle trafficking dysfunction observed in the invasion-associated proteome changes in the BBB. Indeed, we present evidence that invasive disease is associated with a greater degree of altered protein glycosylation. This hypothesis is further supported by the identification of highly significant “synapse” and “plasma membrane” GO terms for altered glycosylated peptides, as many glycosylated proteins are marked for export to the plasma membrane. The function of glycosylation of proteins associated with inflammation remains poorly understood. However, we observed an increase in abundances of glycopeptides derived from proteins involved in neurite outgrowth, including Cntn1, a protein that has been shown to play critical roles in cellular migration in the brain. This finding, paired with the results showing alterations of glycosylated sites in extracellular matrix proteins, suggests that dysfunctional cell-cell association or cellular migration may represent an as-yet-undescribed pathogenic feature of GBS meningitis.

The data presented here suggest that BBB dysfunction associated with GBS meningitis is centered on aberrant ER activity rather than on large-scale disruption of the classical markers of BBB integrity. Future studies can leverage the methods described here to evaluate even earlier time points as well as individual cell type populations to understand the nature, both temporal and cell type specific, of the host-pathogen interaction leading to GBS meningitis. Given the complex makeup of the brain, it is likely that pathogens must engage diverse virulence mechanisms in order to manipulate the various cell types that make up the host system to cause invasive disease. Further studies could also utilize systems biology techniques and isogenic mutants to investigate host molecular changes caused by a key GBS virulence factor, the hemolysin/cytolysin, during BBB perturbation.

To conclude, this study demonstrated the advantages of tissue type-specific molecular profiling over whole-organ studies or those limited to the evaluation of individual protein targets. Investigations of this nature are appropriate for the study of GBS infection in the CNS, as bacteria likely employ a multitude of strategies to circumvent the highly selective vascular barriers separating the blood and the brain. Additional studies using *in vitro* cell culture models could untangle the temporal nature of the various pathways that were found to be altered during GBS infection in this study to determine their importance to GBS pathophysiology. The data accumulated in this study lay the groundwork for future investigations into the molecular interplay between GBS virulence factors and host cell or tissue types. Finally, these methods are broadly applicable, and future systems-level studies concerning pathogens, both bacterial and viral, and their relationship to the host should employ tissue type-specific methods of this nature in order to generate a more complete understanding of the diseased state.

## MATERIALS AND METHODS

### Experimental model and subject details. (i) Animal subjects.

The CD-1 mice used in the study were obtained from Charles River. Male mice aged 8 to 9 weeks were infected as described below. Animals were housed in pathogen-free facilities until infections were performed. Animal experiments were approved by the committee on the use and care of animals at the University of California, San Diego (UCSD), and performed using accepted veterinary standards.

### (ii) Bacteria.

WT and isogenic Δ*iagA* COH1 GBS strains were grown from frozen glycerol stocks in Todd-Hewitt broth (THB) at 37°C. The Δ*iagA* COH1 GBS strain was generated in a previous study (14).

### Mouse model of hematogenous GBS meningitis.

For whole-brain and vascular isolation studies, mice were infected with 1 × 10^8^ CFU of WT COH1 or Δ*iagA* COH1. Bacteria were grown overnight and then back-diluted and grown for 4 h to mid-log phase. Prior to infection, bacteria were pelleted and washed in PBS and serial dilutions were plated on solid medium. PBS was administered as a control.

For CFU enumeration and whole-brain proteomics, 5 animals per group were sacrificed 16 (early), 38 (mid), or 62 (late/moribund) hours postinfection, as these time points corresponded to various stages previously measured by weight loss. Blood was serially diluted for CFU enumeration. Brain hemispheres were homogenized via bead-beating and used for either CFU enumeration or proteomic analysis.

For vascular isolation studies, six animals per infection group were sacrificed 62 h postinfection. Animals were put under general anesthesia by an intraperitoneal (i.p.) injection of a ketamine (100 mg/kg of body weight)/xylazine (20 mg/kg) mixture. Blood was removed by transcardial perfusion of ice-cold Dulbecco’s PBS (DPBS) for 3 min at 4.5 ml/min. All dissections were conducted in cold DPBS. Brains were dissected to exclude the olfactory bulb, optic tract, cerebellum, pons, and medulla. Meninges were removed by rolling brains on Whatman filter paper (hardened grade 50). Choroid plexi were isolated by microdissection and were immediately stored on dry ice. The remaining brain tissue samples were immediately frozen in dry ice prior to vessel isolation.

For the brain imaging cohort, mice were infected with 1 × 10^8^ CFU of WT COH1 (*n* = 3) or Δ*iagA* COH1 (*n* = 4) or were injected with PBS (*n* = 4) and sacrificed 62 h postinfection. Animals were put under general anesthesia by an i.p. injection of a ketamine (100 mg/kg)/xylazine (20 mg/kg) mixture followed by transcardial perfusion of a 0.2 mg/ml EZ-link Sulfo-NHS-biotin–DPBS (Gibco) solution using a Dynamax peristaltic pump for 10 min, followed by 10 min of perfusion with 4% paraformaldehyde–PBS. The flow rate of the pump was adjusted to match the cardiac output of mice (at 4.5 ml/min). Isolated whole brains were submerged in a solution of 30% sucrose for further processing.

### Brain vasculature isolation.

The method used for purification of mouse brain vessels was adapted from an existing protocol (55). Briefly, mouse brains were cut into small pieces manually using a scalpel and subsequently homogenized with an automated Dounce homogenizer (20 strokes, 400 rpm). The pellet was resuspended by addition of dextran buffer, and the mixture was vigorously shaken before centrifugation at 4,400 × *g* for 15 min at 4°C. The supernatant and the white myelin layer formed on top were aspirated, and the remaining myelin was carefully wiped from the tube walls. The pellet containing the blood vessels was resuspended and passed through a glass-bead column. Vessels adhering to the glass beads were collected by washing the beads in a buffer containing bovine serum albumin (BSA). The solution containing purified blood vessels was filtered using a 70-μm-pore-size mesh filter to exclude large blood vessels and a 20-μm-pore-size mesh filter to retain small blood vessels and filter out remaining small debris. Blood vessels from the 20-μm-pore-size filter were used for proteomic analysis.

### Immunofluorescent staining.

Blocking and permeabilization of tissue sections were performed using 50% normal goat serum–0.5% Triton X-100–1.5 M glycine–PBS to quench autofluorescence. Antibodies were diluted in 5% normal goat serum–0.05% Triton X-100–0.15 M glycine at a 1:1,000 concentration and stained overnight at 4°C. Secondary antibodies and BSL were diluted in the same solution at a concentration of 1:1,000 and incubated 2 h at room temperature. When BSL staining was employed, stained sections were incubated in a solution of 4% paraformaldehyde for 10 min following secondary antibody staining. Sections were mounted using Fluoromount-G. For MHC class I analyses, images were taken from three disparate regions of the cortex in all animals. For Vcam1, images were collected from the hypothalamus and from two disparate regions of the cortex in all animals. Image analysis was performed using ImageJ, where background fluorescent was removed by uniformly setting the lower threshold to 65 for MHC class I, to 175 for Vcam1, and to 65 and 100 for MHC class I and Vcam1 BSL images, respectively. Intensity of staining was quantified by measuring the average threshold adjusted intensity over the measured area and normalizing immunofluorescence against BSL values. For Vcam1 staining, the values for each brain region were additionally normalized against the average intensity value.

### Proteomics sample lysis.

Whole-brain tissue and choroid plexus samples were immersed in equal volumes of lysis buffer containing 75 mM NaCl, 3% sodium dodecyl sulfate (SDS), 1 mM sodium fluoride, 1 mM beta-glycerophosphate, 1 mM sodium orthovanadate, 10 mM sodium pyrophosphate, 1 mM phenylmethylsulfonyl fluoride, 1× cOmplete EDTA-free protease inhibitor cocktail, and 50 mM HEPES (Sigma) (pH 8.5). Isolated vasculature samples were immersed in a solution comprised of 1% (wt/vol) n-dodecyl-β-d-maltoside (DDM), 50 mM HEPES (pH 8.5), 0.1 M sodium chloride, 10 mM sodium fluoride, 10 mM β-glycerophosphate, 2 mM sodium orthovanadate, and 10 mM sodium pyrophosphate. Vessels were immersed in a lysis buffer, substituting SDS for DDM. Equal volumes of 8 M urea were added to all samples. Samples were then subjected to probe sonication to ensure complete lysis. Probe sonication was performed using a Q500 QSonica sonicator with a 1.6-mm microtip horn and the pulse setting, which alternated sonication at 20% amplitude for 15 s with 15 s of rest three times.

### Protein extraction and digestion.

Disulfide bond reduction was performed in 5 mM dithiothreitol (DTT) at 56°C for 30 min. Methylation of broken disulfide bonds was then performed in 15 mM iodoacetamide (IAA) in a darkened environment for 20 min. Quenching of the methylation reaction was performed by adding 5 mM DTT and incubating samples in a darkened environment for 15 min (56). Protein from brain sample lysates was then precipitated using a previously described chloroform-methanol precipitation method (62). Protein from endothelial cell samples was precipitated using trichloroacetic acid (TCA). Following protein precipitation, solid-phase pellets were kept on ice to prevent biased sample loss of hydrophobic proteins. Protein pellets were washed with cold acetone following precipitation and centrifuged at 4,000 rpm for 2 min at 4°C. The resultant supernatant was removed, and acetone washes were repeated twice more. Following the final wash, protein pellets were dried at 56°C.

### Protein digestion and TMT labeling.

Dried protein pellets were resuspended in 900 μl of 1 M urea with 50 mM HEPES (pH 8.5). Samples were subjected to vortex mixing for 5 min and sonicated in a water bath sonicator for 5 min to ensure rehydration of the pellets. Digestion of brain protein samples was performed at room temperature overnight with 9 μg sequencing-grade LysC. A second digestion step was performed using 8.6 μg sequencing-grade trypsin at 37°C for 6 h. The trypsin reaction was terminated by addition of 60 μl of 10% trifluoroacetic acid (TFA). Insoluble debris was pelleted by centrifuging the samples for 5 min at 4,000 rpm. Digested soluble peptide was desalted on C_18_ resin columns and dried under vacuum. Lyophilized peptides were resuspended in 1 ml 50% acetonitrile–5% formic acid prior to peptide quantification using a Pierce quantitative peptide assay kit. A 50-μg volume of peptide was separated from each sample for further analysis (57, 58). An internal standard termed a “bridge channel” was prepared from the bEnd.3 peptide isolates by mixing equal amounts of each sample together and separating three aliquots of 50 μg from this mixture. Peptide samples intended for TMT labeling and mass spectrometry-based proteomic analysis were lyophilized overnight under vacuum. These samples were resuspended in 50 μl of a solution of 30% dry acetonitrile and 50 mM HEPES (pH 8.5). TMT reagents were resuspended by vortex mixing for 5 min in a solution of 30% dry acetonitrile and 50 mM HEPES (pH 8.5). Labeling was performed on resuspended peptides by incubation in an 8-μl volume for 1 h at room temperature. Reaction quenching was performed for 15 min by adding 9 μl of 5% hydroxylamine to reaction tubes. After reaction quenching, samples were acidified using 50 μl of 1% TFA. Of note, brain sample label assignment for this experiment was performed as part of a larger experiment performed with a total of 135 mouse brain samples. Sample label assignment was performed such that no two replicates were assigned to the same label channel. Bridge channels for the bEnd.3 proteomics experiment were assigned to the 126 TMT label for all three 10-plexed runs. Following acidification of labeled samples, labeled peptides within each 10-plex were mixed together, desalted on C_18_ resin columns, and lyophilized.

### Sample fractionation.

Choroid plexus and microvessel multiplexed samples were fractionated using spin columns as recommended by the manufacturer (Pierce).

### Liquid chromatography-“tribrid” MS (LC-MS/MS/MS).

Dried fractions were resuspended in a solution of 5% acetonitrile and 5% formic acid. Mass spectrometry-based proteomic data collection was performed using an Orbitrap Fusion mass spectrometer with in-line Easy nano-LC. Fractions were run on 3-h gradients, and the column was washed after each run of the sets of multiplexed fractions. The gradient ranged from 3% acetonitrile and 0.125% formic acid to 100% acetonitrile and 0.125% formic acid over each run. Peptides were separated using an in-house-prepared column with a length of 30 cm, inner diameter of 100 μm, and outer diameter of 360 μm. The column was packed at the front end with 0.5 cm of C_4_ resin (5-μm particle size) and 0.5 cm of C_18_ resin (3-μm particle size). The remainder of the column was packed with C_18_ resin (1.8-μm particle size). Ionization at the source was facilitated by applying 2 kV of electricity through a T-junction connecting sample, waste, and column capillary lines.

MS spectrum acquisition was performed in data-dependent mode with a survey scan range of 500 to 1,200 *m*/*z* and resolution of 60,000. Automatic gain control (AGC) was set to 2 × 10^5^, and the maximum ion inject time was 100 ms. The Top N option was selected, with N set to 10 ions for both MS2 and MS3 analysis.

MS2 data were collected with the decision tree tool. The settings for the decision tree were as follows. Ions with 2 charges were analyzed at between 600 and 1,200 *m*/*z*, while ions with 3 or 4 charges were analyzed at between 500 and 1,200 *m*/*z*. The lower ion intensity threshold was 5 × 10^4^. Selected ions were isolated in the quadrupole at 0.5 Th and fragmented with collision-induced dissociation (CID). Fragment ions were detected in the linear ion trap with a high-scan-rate AGC setting of 1 × 10^4^. Data were subjected to centroid analysis at this stage.

Fragmentation of TMT reporter ions was performed at the MS3 stage using synchronous precursor selection. The 10 precursors chosen at the MS2 stage were fragmented by the use of high-energy collisional dissociation (HCD) fragmentation. Reporter ion detection occurred in the Orbitrap mass spectrometer with a resolution of 60,000 and the lower detection limit set at 110 *m*/*z*. AGC at this stage was 1 × 10^5^, and maximum injection time was 100 ms. Data were subjected to centroid analysis at this stage. Precursor ions 40 *m*/*z* below and 15 *m*/*z* above the MS2 *m*/*z* value were jettisoned at this stage.

### Data processing and normalization.

Spectral matching and filtering were performed using Proteome Discoverer 2.1 software (56, 59, 60). Spectral matching was performed using the Uniprot Mus musculus reference proteome downloaded on 2 July 2018 and appended to the COH1 GBS reference proteome downloaded on 6 July 2018.

For proteomic analysis, the SEQUEST algorithm was used for decoy database generation. Precursor ion mass tolerance was set to 50 ppm, and fragment ion mass tolerance was 0.6 Da. The enzyme was set as trypsin, and two missed cleavages were allowed. The peptide length range was 6 to 144 amino acids. One dynamic modification was used, methionine oxidation (+15.995 Da). Static modifications included isobaric tandem mass tags at the N termini and on lysine residues (+229.163 Da) and carbamidomethylation of cysteines (+57.021 Da). Filtering of spectra was performed in Percolator at the peptide and protein levels against the previously generated decoy database. For glycoproteomic analysis, the processing node was modified to include Byonic. Within the Byonic node, the built-in N-glycan mouse plasma database was specified as a “Dynamic—Rare” setting.

Following search completion, data were filtered for high peptide spectral match (PSM) confidence and PSM disambiguity/selection. For proteomics, filtered PSM quant data were summed to the protein level, and for glycoproteomics, filtered PSM quant data were summed to the unique-glycopeptide level. Whole-brain and choroid plexus proteomic data were normalized against the average relative abundance for a given protein divided by the median of all average values. A second normalization step was performed by normalizing the average-normalized values against the median values for each TMT channel divided by the median of all TMT channel medians. Brain microvessel proteomic data were normalized against the bridge channel values for each protein divided by the median of all bridge channel values. A second normalization step was performed as described above.

### GNPS analysis.

Mass spectrometer-generated .raw files were converted to .mzML files using MSConvert. GNPS spectral networking was run on files using a precursor ion tolerance of 0.6 Da and a fragment ion tolerance of 0.02 Da. Cosine score threshold was set to 0.7, and the minimum matched-fragment ion value was set to 6 ions.

### Statistical methods.

For CFU enumeration, statistical significance was determined through one-way analysis of variance (ANOVA) with Tukey’s multiple-comparison test. For determinations of staining intensity in immunofluorescence studies, one-way ANOVA was used to determine statistical significance. π score data (proteomics π > 1; glycoproteomics π > 0.5) were used to evaluate differences in protein abundance from proteomics data, with *P* values derived from the use of Student's *t* test with or without Welch’s correction (61). K-means clustering was performed using Morpheus (https://software.broadinstitute.org/morpheus/). GO analysis was performed using g:Profiler (http://biit.cs.ut.ee/gprofiler/gost), and term enrichment was determined by Benjamini Hochberg-corrected *P* value.

### Figure generation.

Histograms and volcano plots were generated using GraphPad PRISM 8. Bubble plots were generated in R using the ggplotly function. Networks were generated using String-db (https://string-db.org/cgi/input.pl?sessionId=treosIb26CwC&input_page_show_search=on). String-db interaction networks were imported to Cytoscape for final figure generation. Heat map generation was performed using Morpheus (https://software.broadinstitute.org/morpheus).

### Data availability.

Raw mass spectrometry data can be found in the MassIVe spectral repository and are available at ProteomeXchange under the PXD013946 identifier for whole-brain data, PXD013917 for choroid plexus data, and PXD013936 for brain vessel data.
