# Supplementary figures and images for: Multidimensional Proteome Profiling of Blood-Brain Barrier Perturbation by Group B Streptococcus
Source: mSystems. 2020 Aug 25;5(4):e00368-20. doi: 10.1128/mSystems.00368-20 (PMC7449606; doi:10.1128/mSystems.00368-20)

Figure S1

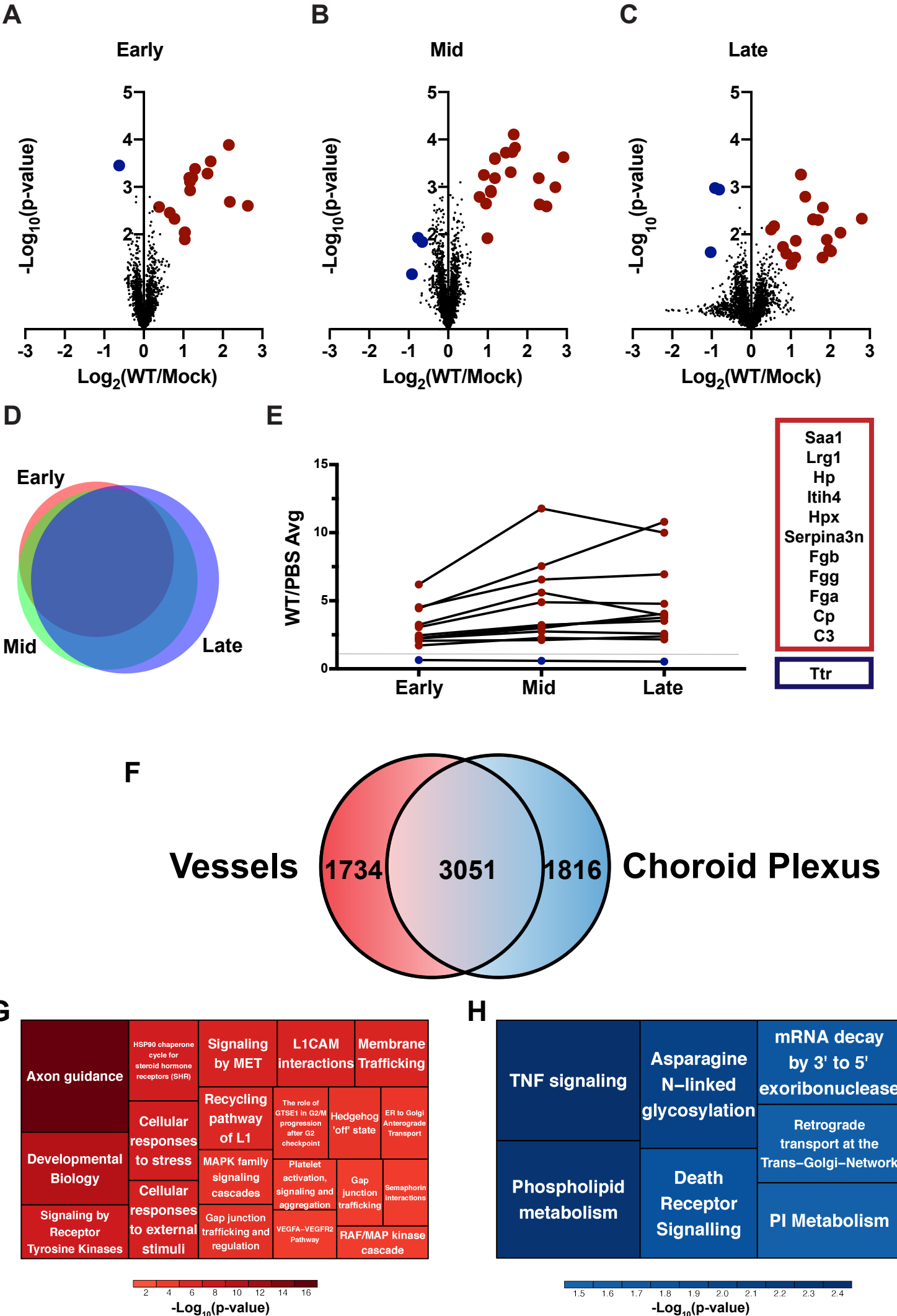

Supplement: FIG S1 [file mSystems.00368-20-sf001.pdf]

Figure S2

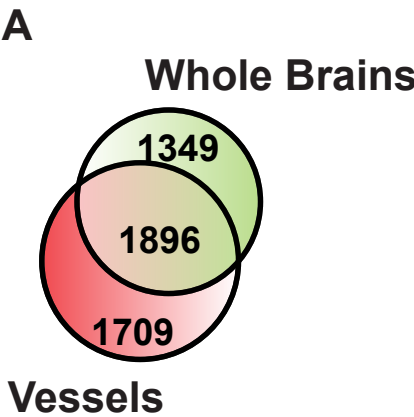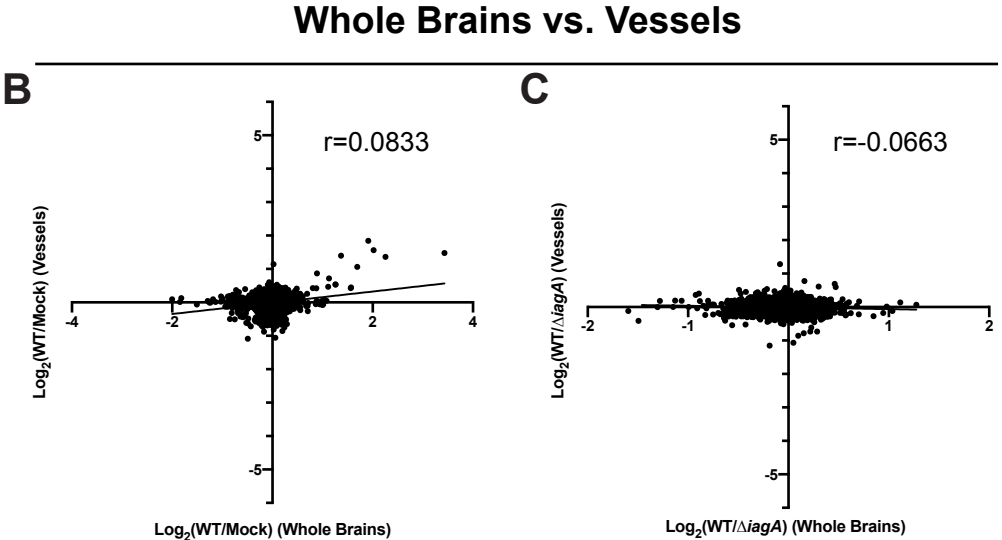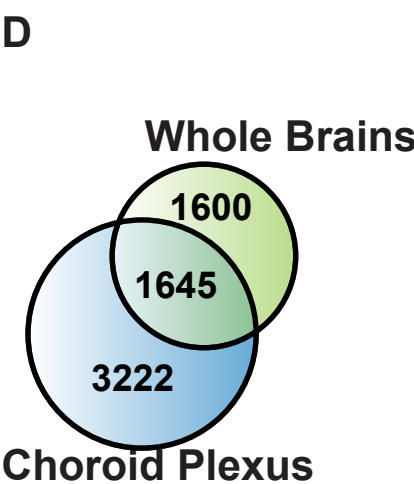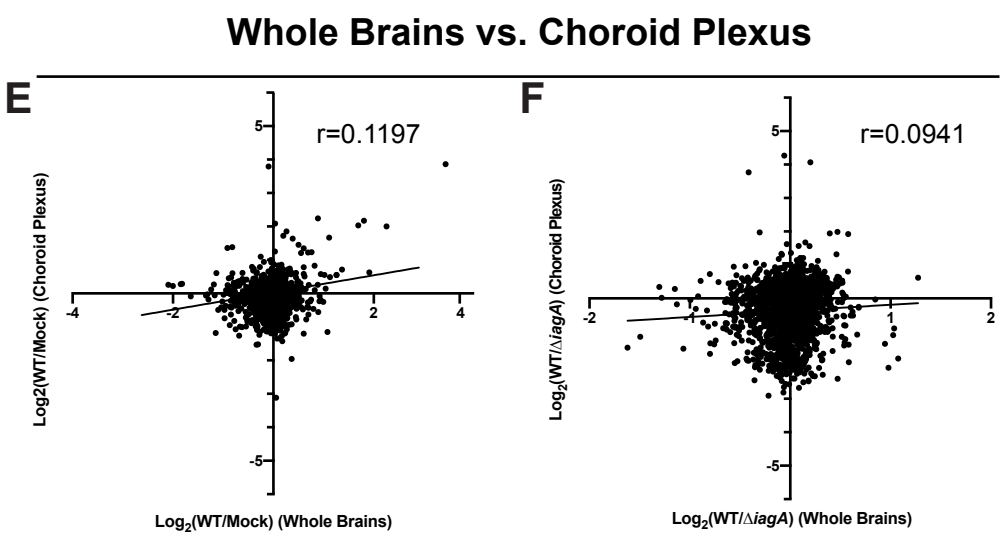

Supplement: FIG S2 [file mSystems.00368-20-sf002.pdf]

Figure S3

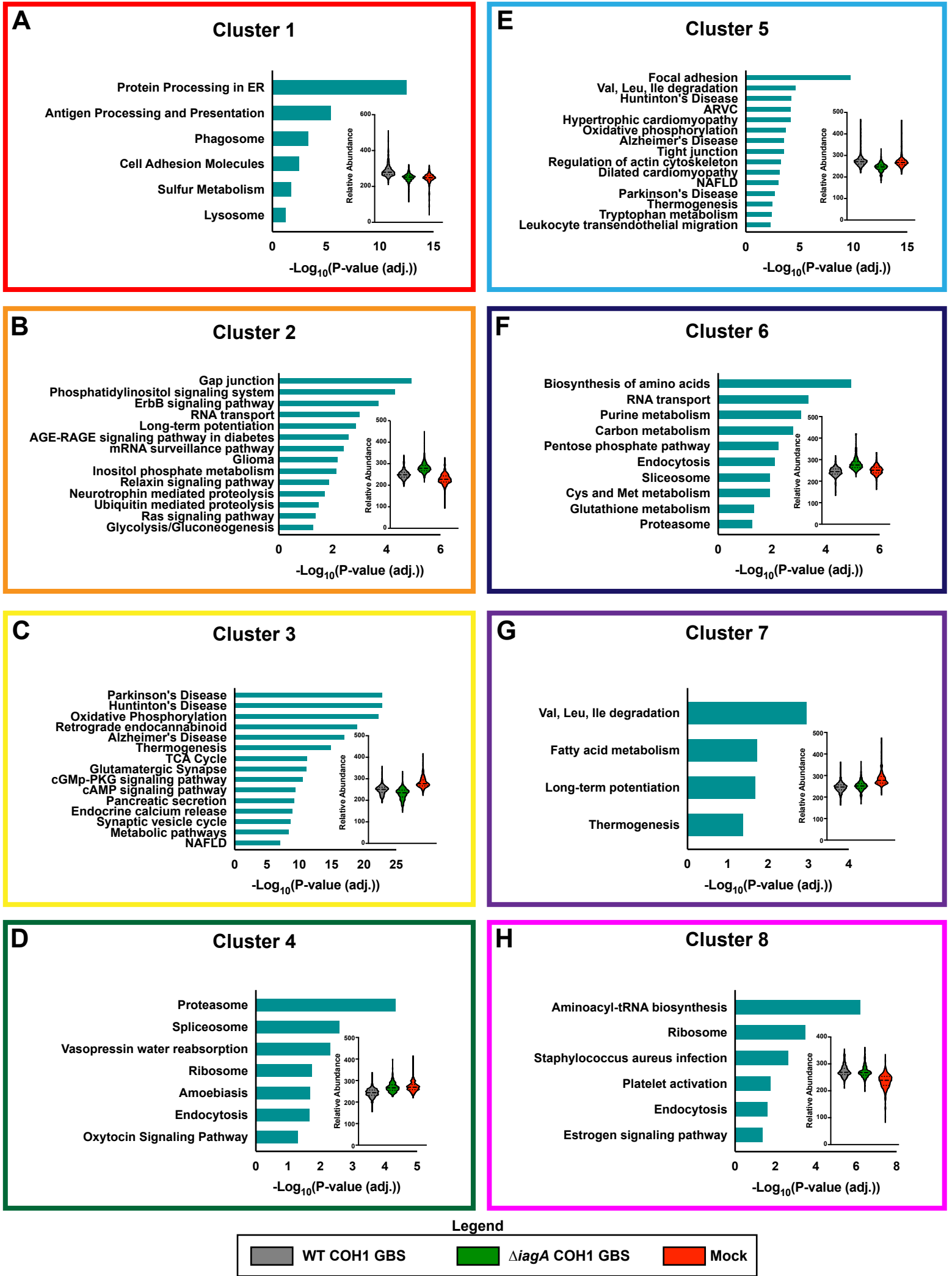

Supplement: FIG S3 [file mSystems.00368-20-sf003.pdf]

**Figure S4**

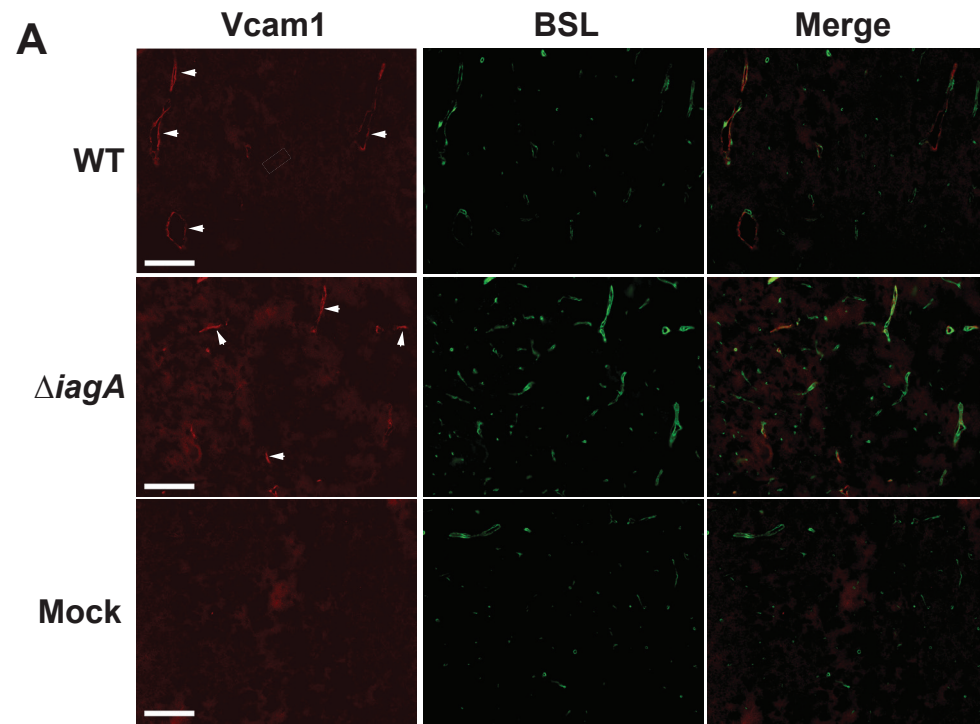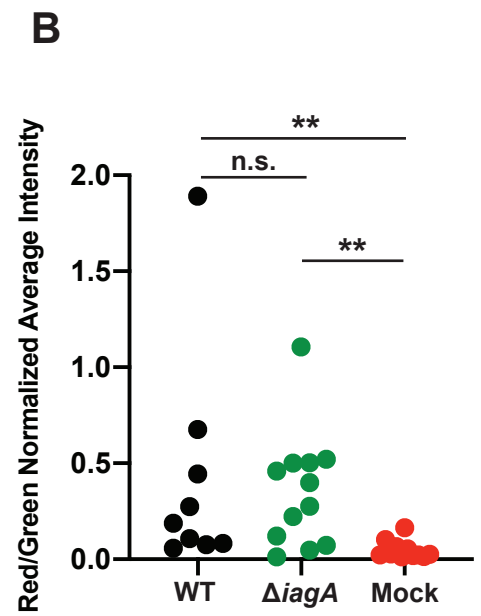

Supplement: FIG S4 [file mSystems.00368-20-sf004.pdf]

Figure S5

A

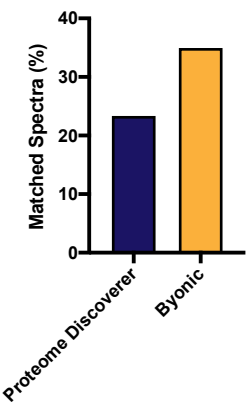

B

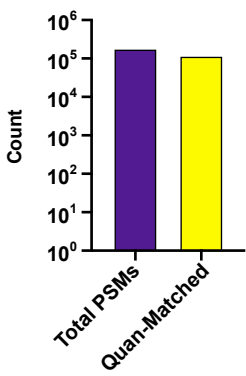

C

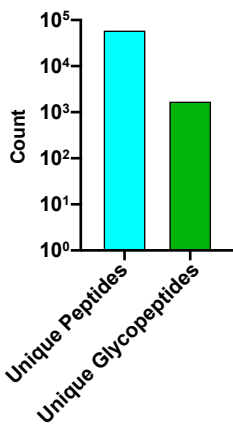

Supplement: FIG S5 [file mSystems.00368-20-sf005.pdf]
